# Artificial Grammar Learning of Melody Is Constrained by Melodic Inconsistency: Narmour's Principles Affect Melodic Learning

**DOI:** 10.1371/journal.pone.0066174

**Published:** 2013-07-09

**Authors:** Martin Rohrmeier, Ian Cross

**Affiliations:** 1 Massachusetts Institute of Technology, Cambridge, Massachussetts, United States of America; 2 Centre for Music and Science, Faculty of Music, University of Cambridge, Cambridge, United Kingdom; UNLV, United States of America

## Abstract

Considerable evidence suggests that people acquire artificial grammars incidentally and implicitly, an indispensable capacity for the acquisition of music or language. However, less research has been devoted to exploring constraints affecting incidental learning. Within the domain of music, the extent to which Narmour's (1990) melodic principles affect implicit learning of melodic structure was experimentally explored. Extending previous research (Rohrmeier, Rebuschat & Cross, 2011), the identical finite-state grammar is employed having terminals (the alphabet) manipulated so that melodies generated systematically violated Narmour's principles. Results indicate that Narmour-inconsistent melodic materials impede implicit learning. This further constitutes a case in which artificial grammar learning is affected by prior knowledge or processing constraints.

## Introduction

Implicit learning constitutes the core process for human enculturation in respect of complex forms of communication such as music or language [1]–[4]. Humans need to have access to a large amount of structural musical knowledge in order to make sense of the music of their culture. Despite having very little or no explicit or formal musical training, most members of a community possess competence of the music of their society [5]–[7]. Musical knowledge, like native language knowledge, is largely implicit, being represented without awareness of its complex structures and incidentally acquired through long-term interaction with music. Accordingly, musical competence and knowledge of stylistic structures is assumed to be acquired during musical interaction and implicit learning constitutes a central process in musical enculturation [8]–[10]. At present empirical evidence is ambiguous with respect to whether statistical melodic learning in patients, who suffer amusia, is intact [11] or impaired [12], [13].

A number of experimental studies have studied incidental, statistical or implicit learning of musical structure under different paradigms (see [8] for a review). Saffran and colleagues studied statistical learning of “tone words” from a continuous monophonic and isochronic melodic stream in the context of segmentation [14]. Several studies explored learning of melodic structures generated by finite-state grammars [15]–[18]. Other studies used musical structures of a greater complexity. Kuhn & Dienes used self-similar melodies employing a bi-conditional grammar (in which the second half of a stimulus would be the inversion of the first half) [19], [20]. In another study, Dienes and Longuet-Higgins used a 12-tone serialist paradigm to construct 12-tone rows with a structure in which the second half of a row would be a serialist transformation (transposition or inverse retrograde) of the first half [21]. Rohrmeier and Cross found that complex harmonic sequences modeled from a recursive context-free grammar were implicitly learned [22], matching a finding from a comparable set-up in artificial language learning [23], [24].

While these and other studies suggest that knowledge of different musical features is acquired and represented implicitly, they mostly focus on various musical features or structural complexity. However, much research in statistical or implicit learning (of music) does not investigate constraints or effects of pre-processing on implicit artificial grammar learning (cf. [25]). The aim of this study is to explore whether implicit learning of melodic structure is affected when melodies of identical complexity differ largely from common structures employed in melodies across cultures. The results may shed light on effects of pre-processing or priming and learnability of such melodies with further implications for music cognition. Moreover, such an exploration further entails implications for the general field of implicit learning.

## Background

**Figure 1:**
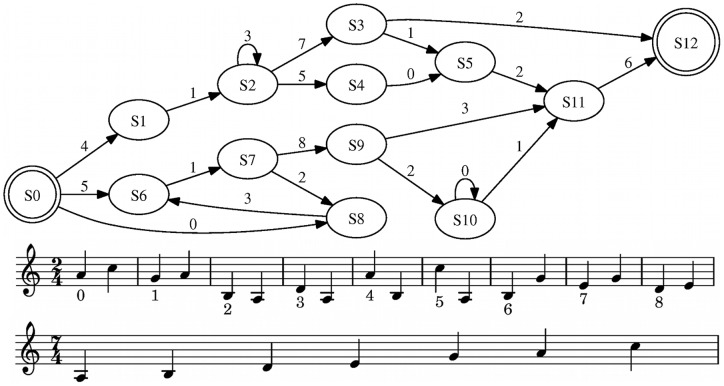
The finite-state grammar, terminal tone pairs and the scale used in this study. The grammar is identical to the one used by [15].

A study by Rohrmeier, Rebuschat and Cross [15] found that participants were able to acquire new melodic patterns with high efficiency, when these conformed to common melodic principles. The materials were generated from a finite-state grammar and its *terminal symbols* (i.e. the alphabet used to generate sequences; in this case, tone pairs as in Fig. 1) were intentionally designed in a way so that they would produce coherent, acceptable (yet not formally tonal) melodies for the participants. The purpose of the present study was to investigate the extent to which the learning of melodic patterns would be affected when the melodic structures frequently contravene common and ubiquitous principles of melodic structure as formalised in the principles proposed by Narmour [26]. Accordingly, the aim of the experiment was to manipulate the materials used by Rohrmeier et al. in a systematic way, so that they would maximally violate the quantified versions of Narmour's principles [27], [28] of registral direction, registral return, intervallic difference, proximity, closure and consonance.

Narmour's Implication-Realization theory (IR, [26], [29], [30]) describes core properties of melodic structure and extends ideas by Meyer [31], [32]. The basic components of the theory have been summarised in many places [27], [33], [34] and will therefore be described only briefly. The IR theory characterises melodic expectation with respect to the tendencies with which melodic *implicative* intervals proceed to specific subsequent *realised* intervals. It postulates a bottom-up and top-down system of melodic perception, in which the former is assumed to be innate and universal, and the latter to be learned through interaction with music. Schellenberg as well as Krumhansl proposed a simplification and quantification of Narmour's theory based on five principles (see [28], [27], and below). While Narmour argued the principles below to be universal and innate [26], computational accounts by Pearce & Wiggins argued that they could be accounted for on the basis of computational n-gram learning (n-grams refer to small chunks of the size n) [35].

### 

#### Registral directon

Small intervals tend to be continued in the same direction whereas large intervals tend to be continued in the opposite direction.

#### Registral return

This principle describes an implication for a realised interval to return to the same pitch or neighbouring pitches (+/−2 semitones), when it changes the direction of the implicative interval.

#### Intervallic difference

Small intervals imply a realised interval of the same size (+/−2 semitones if the direction changes, +/−3 semitones if the direction is the same). Large intervals imply a realised interval of a smaller size (at least 3 semitones smaller).

#### Proximity

The interval between any two tones is in general small (5 or fewer semitones).

#### Closure

Involves either a change in registral direction, or a large implicative interval being followed by a smaller interval (smaller than 3 semitones for the identical registral direction or smaller than 2 semitones for a different registral direction), and the realised interval is small.

#### Consonance

Models whether the interval between two adjacent notes is consonant, based on an empirically derived weighting vector for the 12 chromatic intervals in the octave [36].

Whereas [26] had outlined and exemplified his theory with a large number of musical examples from a wide range of styles and cultures, a number of empirical studies tested the relevance of the principles for melody perception. Cuddy and Lunney [37] let participants rate a large set of 2-interval patterns and found that responses largely conformed to the quantified version of Narmour's principles, particularly with respect to *intervallic difference*, *registral return* and *proximity* and found no effect of musical training. Krumhansl [36] carried out a similar study which employed a larger set of melodic two-interval patterns. Similarly, she found some support for the features of *proximity*, *registral direction*, *registral return*, but not *intervallic difference* nor *closure*. Another study by Krumhansl further explored the validity of the theory in terms of real music fragments (British and Chinese folksongs and Webern lieder) using a tone continuation paradigm and found support for all five principles (except *intervallic difference* for Webern lieder) [27]. In subsequent research Schellenberg [28], [34] showed that the quantification of Narmour's model was still redundant and could be further simplified to only two principles: a revised version of *proximity* and a principle of *pitch reversal* combining *registral direction* and *registral return*. This simplified model had no loss in explanatory power for the experimental data of Cuddy and Lunney [37] as well as Krumhansl [27].

Concerning the cross-cultural extension of the principles, empirical findings are ambiguous to some extent. Eerola and colleagues [38]–[40] found that the melodic expectancy of a group of South-African healers could be well characterised by Narmour's principles. Carlsen, however, found cultural differences in terms of melodic expectation [41]. Two further studies [42], [43] questioned the cross-cultural validity of the (simplified) set of principles and found that the factors from [36] matched the data better than the versions by Schellenberg [28], [34]. On the contrary, Schellenberg and colleagues found that the behaviour of adults and infants was well explained by Schellenberg's revised model [44].

Despite the theoretical disputes, Narmour's principles have been shown to be instantiated empirically in large sets of musical pieces and to be relevant for (at least Western) perception of melody. This motivates a study to explore how learning of melodies is affected when stimuli are used that do not conform to these core melodic principles.

## Materials and Methods

### Participants

The experimental protocol was approved by the research governance procedures of the Centre for Music & Science, Faculty of Music, University of Cambridge. 31 adults (14 women, 17 men, mean age 23.0 years) participated in the experiment. All participants provided informed consent prior to the experiment. The experimental group had 15 musicians and 16 nonmusicians. Musician participants all played their instrument(s) actively, had an average of 13.5 years of music lessons and practised/performed 9.7 hours per week on average. Nonmusician participants did not play their instrument(s) any more, had never played an instrument or had only played for a short period. In total, nonmusicians had an average of 0.7 years of music lessons and practised/performed 0.4 hours per week on average. For the sake of comparability with our study on learning of Narmour-consistent melodies [15], the same subject pool was used for the present study. Musician participants were recruited from the Faculty of Music and nonmusician participants were recruited on campus, both at the University of Cambridge. None of the participants had participated in the prior baseline study [15], which used 11 musicians and 11 nonmusicians respectively. In that study, musician participants all played their instrument(s) actively, had an average of 11.7 years of music lessons and practised/performed 6.2 h per week on average. All nonmusician participants did not play music actively (0 h per week), had not practiced an instrument for 2.8 years on average and had stopped practising (if they had played) for 9.1 years on average.

### Materials

The grammatical stimuli consisted of 33 different melodies between 8 and 30 tones generated from a regular grammar (see Fig. 1). To investigate whether stimulus learning would derive from mere sequence memorisation or induction of some underlying structure, 17 of these melodies were employed for the learning and testing phase (“old-grammatical”) and 16 remaining melodies were only used for the testing phase (“new-grammatical”). Five types of ungrammatical stimuli were used. First, error types 1–3 were intended to test whether participants would detect different forms of random disorder in the melodic sequences: most simply, error type 1 consisted of entirely random sequences of the terminal tone pairs. In contrast, error type 2 sequences employed correct transitions between terminal tone pairs, but their overall sequence would be random. Hence, for each ungrammatical sequence every single transition between two terminal tone pairs was part of one grammatical sequence but longer sequences of three or more tone pairs were not. Error type 3 featured correct subsequences for possible stimulus beginnings and endings (according to the finite state grammar; “anchor positions”) with random state sequences (like error type 1) in between; in comparison to error type 1 detecting these structures would require that participants were attentive to more than just anchor positions [45], [46]. In contrast to error types 1–3, error types 4 and 5 stimuli were intended to be very similar to grammatical structures. In the case of error type 4, two halves of grammatical sequences from different pathways were combined, e.g. a stimulus may begin with a grammatical subsequence of the upper pathway of the grammar representation (Fig. 1) and continue with a subsequence of the lower part until the end. Hence, the sequence would be very similar to a grammatical sequence except for the position where the two halves connect and the overall organisation of the sequence. In contrast, error type 5 sequences were intended to only deviate minimally from grammatical sequences by swapping two adjacent terminals or deleting a terminal. It was hypothesised that the numerical ordering of the five error types would reflect the degree of difficulty of the recognition of the stimulus. There were 33 ungrammatical stimuli and their lengths matched the lengths of the grammatical stimuli so that stimulus length would be no indicator of grammaticality. There were six structures for each of error types 1 and 2, and seven structures for each of the remaining error types. Altogether, the testing set consisted of three types of stimuli: old-grammatical, new-grammatical and ungrammatical stimuli (of five different types). This grammar as well as the set of grammatical state sequences generated from the grammar and the ungrammatical sequences are identical with the materials used in the baseline experiment [15].

As outlined above, the aim of the present study was to manipulate the terminals in such a way that the melodies produced by the grammar would frequently violate Narmour's principles while the state sequences would be identical to the baseline study. In order to ensure that the resulting new pitch structures would not alter the underlying grammar, the changes were realised as isomorphic one-to-one mappings from the original set of pitches employed to a new set of pitches (see figure 1). In this way, grammar and terminal tone pairs would remain identical, but the actual surface pitch sequences would be different. Hence the abstract n-gram structure of the two sets of sequences would remain identical and indistinguishable for a computer (such as a chunking model). In order to systematically specify a mapping to generate melodies that frequently violate Narmour's principles, an algorithmic method was employed which selected mappings that were strongly inconsistent with Narmour's principles using a score system. In order to ensure that solutions were possible which did not favour the occurrence of small melodic intervals, the range/tessitura of the pitches employed was augmented from an octave to a tenth (Figure 1) which would allow melodies to break the principles of *registral return* or *registral direction*.

For the computational searching of a good one-to-one mapping for the purpose of the experiment, first, all 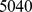 one-to-one mappings were computed. Subsequently, a numerical score of how well the structures conformed to the quantified form of Narmour's principles was computed for each of the melodies in each of the resulting melody sets. Hence each one-to-one mapping solution was characterised by the overall score of its set of melodies. The final solution was selected (manually) from the top ranking solutions. For the computation of the score for each mapping, the MIDI Toolbox for MATLAB [47] implementation of the quantification of Narmour's principles according to [27], [28] (including the additional consonance factor suggested by [27] which encodes whether the realised interval is consonant or dissonant) was employed. Accordingly, the mean (predictability) score for the factors *registral return*, *proximity*, *intervallic difference*, *closure*, revised forms of *registral direction* [28], and *consonance* [27] were computed for the whole set of grammatical and ungrammatical melodies for each mapping. Each mapping was represented through a vector of six mean values for the six different factors. The competitive score of each mapping was computed as the unweighted sum of the z-scores of each of its six component factor values (which were each computed in comparison to the values of all other mapping solutions for the same factor). The mean z-score for the unchanged baseline set was 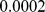, the mean z-score for the chosen solution was 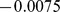. Figure 1 displays the old and the new set of terminals. All stimuli were computationally generated and rendered from MIDI using a synthesised instrument (piano) and applying a 330 ms inter-onset interval per note and a MIDI velocity (loudness) of 100.

### Procedure

For the implicit learning experiment the same procedure was used as in the baseline study [15] (including the same computers, headphones and rooms) and is described fully in the following text. The experiment consisted of a learning phase and a testing phase. The learning phase was not announced as such. Participants were exposed to the stimuli under incidental learning conditions by means of a tone-counting task. Participants were also not informed that they would be tested afterwards. Participants listened to three blocks of all 17 old-grammatical melodies in randomised order and reported the number of tones in each melody. As this task was difficult for some participants, participants could repeat each of the 51 melodies as often as they wanted. The testing phase presented all 66 grammatical and ungrammatical stimuli in randomised order. Participants responded to each stimulus with forced-choice familiarity ratings (familiar vs. unfamiliar) and subsequent binary confidence judgments (high vs. low confidence). A post-test debriefing session required participants to verbalise any rules or regularities they might have noticed. The instructions emphasised that the task was not easy and that participants should follow their intuition.

### Data analysis

Each trial was coded for accuracy based on familiarity ratings: responses for old-grammatical or new-grammatical melodies were coded as correct when chosen as *familiar* and ungrammatical melodies were coded as incorrect when chosen as *familiar*. Since the total number of grammatical and ungrammatical stimuli was identical, the chance level of performance is 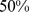. Planned analyses involved comparing the single group performance (in terms of accuracy) against chance performance for the types of grammatical and ungrammatical stimuli, as well as comparing the performance of the present experimental group with the results from the baseline study with respect to the types of grammatical and ungrammatical stimuli. For purposes of comparison, this second analysis was performed in analogy to the baseline analysis in [15].

## Results

Two outlier subjects whose performance differed from the mean of the group by more than two standard deviations were excluded from the analyses (Both participants were more than 2 SD above the mean performance. One participant had reported difficulties with the tone-counting task and had about 5.5 times the median amount of exposure. The exposure of the second outlier was above the median, but there is no clear basis for inferring which factor may have caused the exceptionally high performance in this latter case.) The number of stimulus repetitions in the learning phase had no correlation with performance or confidence levels (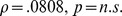 and 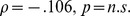 respectively).

### Familiarity judgments

**Figure 2:**
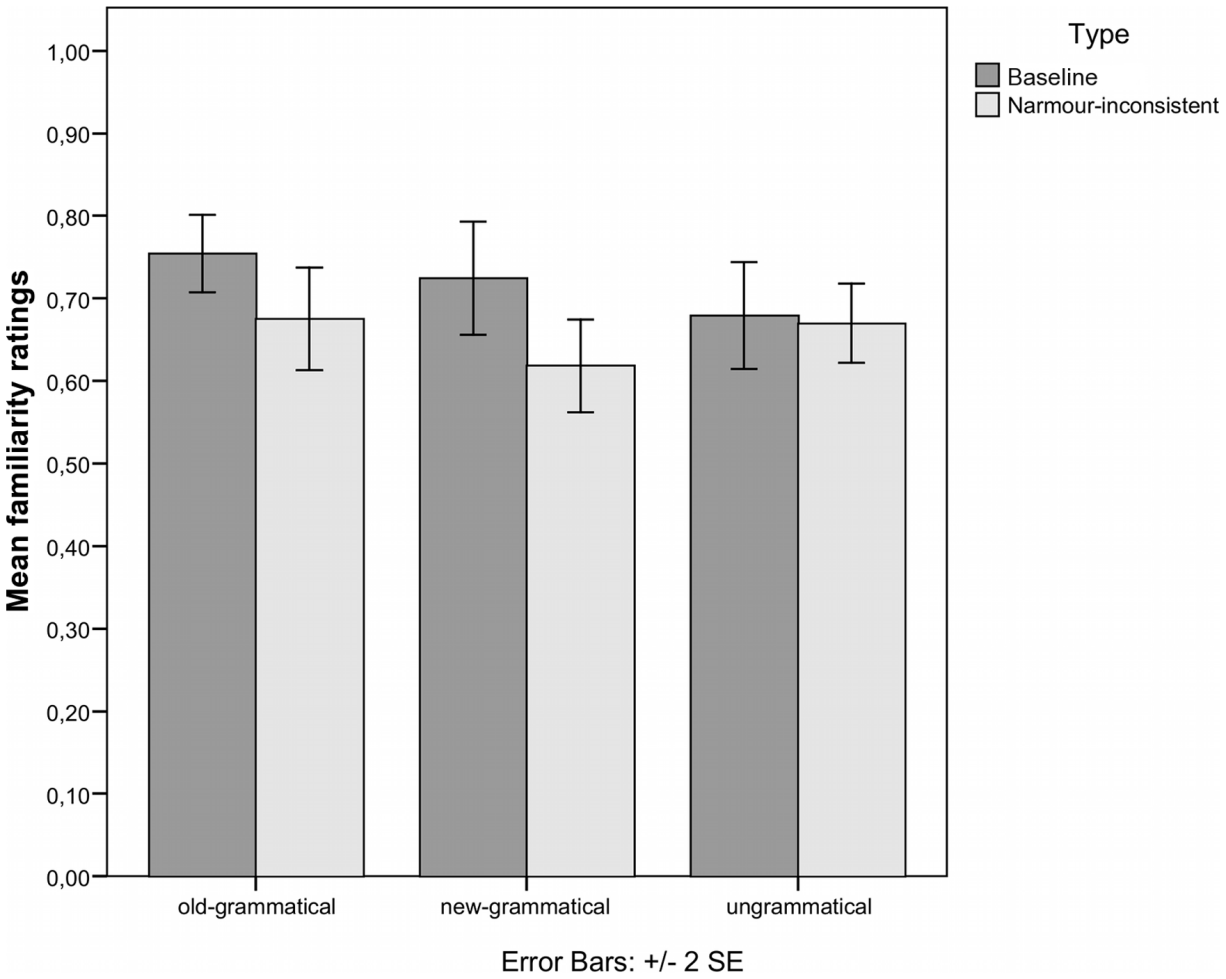
Performance for the baseline [15] and Narmour-inconsistent group. The graph displays the mean familiarity judgment accuracy for old-grammatical sequences (grammatical sequences used in the learning and the testing phase), new- grammatical sequences (grammatical sequences only used in the testing phase) and ungrammatical sequences.

Planned one-sample t tests show that the performance for familiarity judgments differs significantly from chance for old-grammatical stimuli, new-grammatical stimuli and ungrammatical stimuli except error type 5 after applying the sequential Bonferroni procedure (cf. [48], [49]; see Table 1 and Figure 2). The participants in the present experiment performed worse than the experimental group from [15] with respect to overall performance, 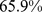 vs. 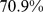 respectively. A 2-by-2 ANOVA with group (baseline (experimental group, [15]) vs. experiment 1) and musical training (musicians vs. nonmusicians) as between-subject variables and grammaticality (composed of performance for old-grammatical, new-grammatical and ungrammatical structures) as within-subject variable found a highly significant effect of group 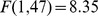, 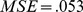, 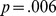, no significant effect of musical training 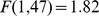, 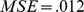, 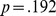, and no significant interaction between group and musical training 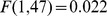, 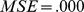, 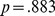. There were no further significant interactions or within-subjects effects, all 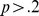. Tests of simple within-subjects contrasts found a significant difference with respect to old-grammatical vs. new-grammatical stimuli, 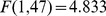, 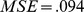, 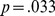.

**Table 1:** Accuracy results.

	Overall accuracy	grammatical			ungrammatical					
		(all)	old-gram	new-gram	(all)	Error type 1	Error type 2	Error type 3	Error type 4	Error type 5
Mean	.659	.648	.675	.619s	.670	.874	.707	.645	.601	.557
SD	.072	.142	.168	.151	.129	0.159	0.234	0.193	0.207	0.196
*t*(28)			5.64	4.24		12.70	4.76	4.05	2.63	1.56
Sig. (2-tailed)			.000	.000		.0000	.0001	.0004	.014	.131

Performance for the different stimulus types in the present experiment and one-sample t tests against 0.5 chance level.

The new group was able to distinguish grammatical from ungrammatical stimuli above chance. Crucially, the performance was significantly lower than the performance for the group in [15]. The contrast further indicated that there was a significant difference between old-grammatical and new-grammatical stimuli, which indicates that participants were better for materials that they had heard before in the training phase.

These findings suggest that the change of the terminal symbols violating Narmour's rules affected the overall implicit learning performance, even though the melodies were still learnable. This suggests that melodic structures that violate common melodic principles are harder to learn or to recognise. There was no significant difference in performance between musicians and nonmusicians. This indicates that any effect of musical training lies within the 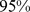 confidence interval 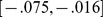 for the difference in performance. This result suggests that musical experience (and hence long-term active engagement in listening to and performing a large repertoire of Narmour-consistent materials) had little or no impact on the learning outcome.

### Error types and type of knowledge

**Figure 3:**
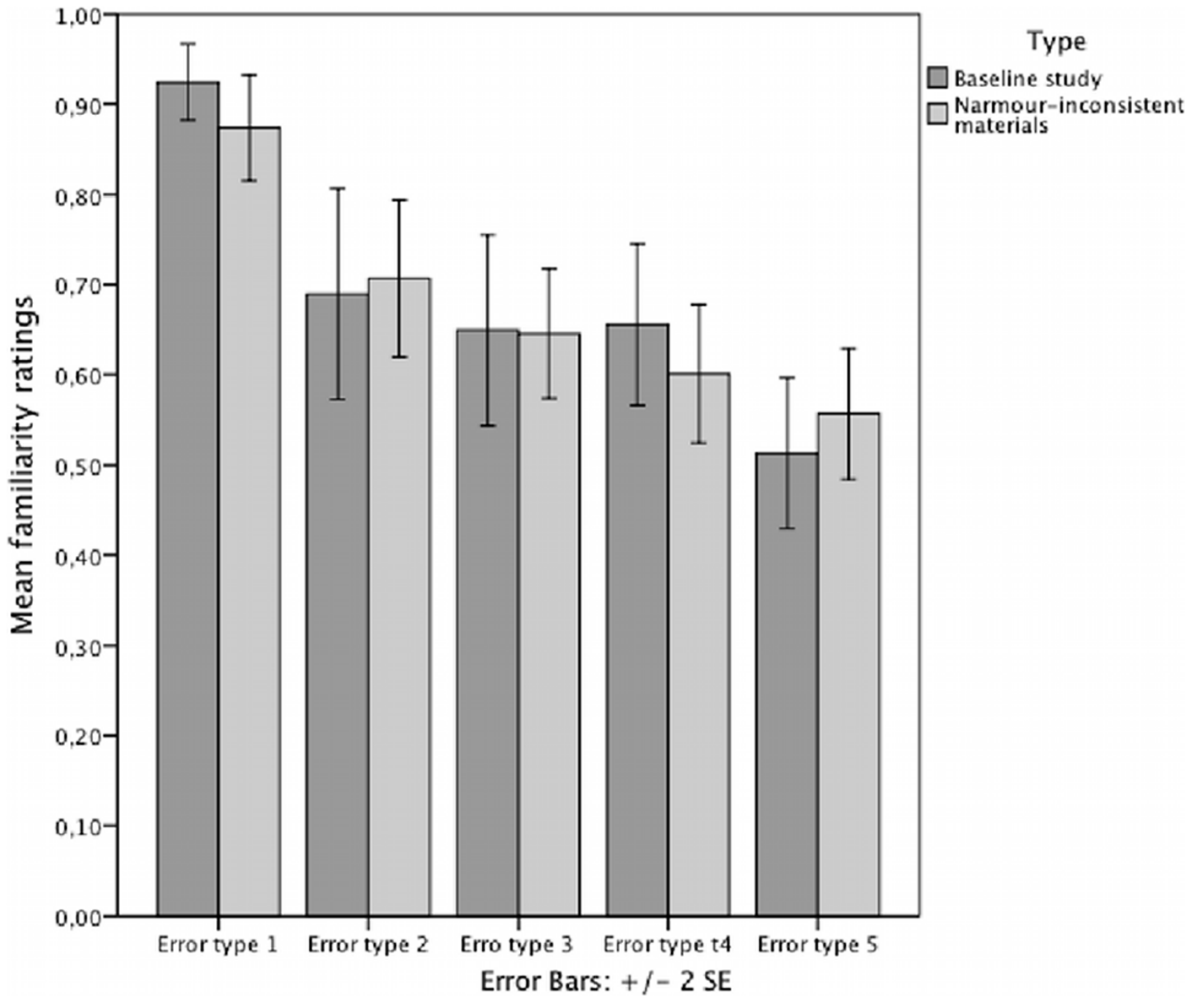
Performance for the five error types in the baseline [15] and Narmour- inconsistent group. The graph displays the mean familiarity judgment accuracy of both groups for the five error types.

One-sample t tests showed that the present experimental group performed above chance for error type 1–4 (applying the sequential Bonferroni procedure, see [48], [49]). Table 1 and Figure 3 represent the results. A 2-by-2 ANOVA with group (experimental vs. baseline) and musical training (musicians vs. nonmusicians) as between-subject variables and error-type (composed of the performance for error-type 1, 2, 3, 4, 5) as within-subject variable found no significant effect of group 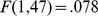, 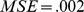, 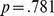, no significant effect of musical training 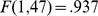, 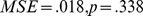, and no significant effect of group and musical training 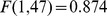, 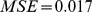, 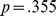. This suggests that there was no statistically significant difference between the performances for ungrammatical stimuli between both groups. Test of repeated within-subjects contrasts were significant for error type only (applying the sequential Bonferroni procedure), in terms of the differences between error type 1 vs. error type 2, 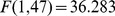, 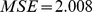, 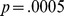, and error type 4 vs. error type 5, 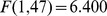, 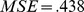, 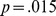. This suggests that for both experiments error type 1 was significantly better recognised than error type 2, and that error type 4 was significantly better recognised than error type 5.

A subsequent analysis explored the extent to which participants' endorsements were potentially based on these differences in fragments for the different stimuli similarly to the methodology employed by [21]. First we computed the average chunk strength for each stimulus. Chunk strength is defined as the count of the number of times each chunk of size 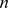 (also referred to as n-gram) occurred in the training stimuli. The average chunk strength is the mean chunk strength for a stimulus. For each participant, a multiple logistic regression using average chunk strength for different chunk sizes 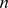 and grammaticality as predictors for the participant's responses for each stimulus was computed for either pitch or interval sequences. T tests comparing the beta coefficients of these predictors against 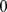 (Table 2) found that grammatical structure proved not to be a significant predictor of participants' responses in both pitch and interval cases. However, bi- and trigrams were found to predict responses across participants in the pitch case. Moreover, 7- and 8-grams were found as predictors in the interval case. This result suggests that participants mainly acquired and applied knowledge about the small fragments as well as larger subsequences for their classification responses. The findings show that there is no evidence that participants have acquired a form of rule or grammatical knowledge beyond fragments in their mental representation of the materials.

**Table 2:** Logistic regression analyses.

	Pitch				Interval			
	Mean	SD	Sig.	*T*(28)	Mean	SD	Sig.	T(28)
intercept	4.354	11.207	0.046	2.092	1.495	3.197	0.018	2.518
1-grams	0.032	0.331	0.602	0.528	0.008	0.136	0.748	0.324
2-grams	−0.359	0.625	0.005	−3.092	−0.115	0.390	0.123	−1.589
3-grams	0.219	0.555	0.043	2.123	0.024	1.050	0.904	0.122
4-grams	0.207	1.143	0.338	0.974	0.046	2.049	0.904	0.122
5-grams	−0.889	2.537	0.070	−1.888	0.216	3.666	0.754	0.317
6-grams	0.918	3.573	0.178	1.383	0.732	3.657	0.290	1.078
7-grams	−0.987	2.865	0.074	−1.855	−3.806	6.251	0.003	−3.279
8-grams	0.941	2.490	0.052	2.035	3.298	4.217	0.000	4.211
grammatical structure	−0.540	1.479	0.059	−1.967	−0.526	1.506	0.071	−1.881

Results from logistic regression analyses [21] across participants using chunks and grammaticality (coded as 1 for grammatical and 0 for ungrammatical) as predictors for participant responses for the cases of pitch and interval structure.

### Confidence ratings and debriefing

As in [15], confidence ratings were analysed by computing Type 2 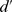 values for proportions of confident correct (hits) and confident incorrect (false alarm) responses [50]. The mean 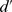 values for the experimental group 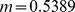; 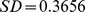; 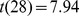; 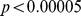; were significantly above zero, indicating that the participants did possess and apply explicit judgment knowledge about their familiarity judgments [51]. In the debriefing session no participant could verbalise any significant rules or regularities in stimulus structures. Accordingly the findings of this experiment are analogous to the results of the baseline experiment [15] in which participants were found to possess in part explicit judgment knowledge and to know when they were right in their responses. Altogether, results indicate that the participants became aware of the incidentally acquired knowledge that was guiding their familiarity judgements.

## Discussion

The present results suggest that the change of the melodic surface structure to violate Narmour's rules affected the overall learning performance of the experiment, yet nonetheless the group still performed above chance. Accordingly, melodic structures which violate common melodic principles seem to be harder to be learned or processed than those which do not. This finding raises several potential explanations and bears consequences with respect to general artificial grammar learning, musical acquisition and processing as well as the emergence of musical structures.

The present results link up with further evidence from musical statistical learning studies [8], [52]–[54]. Using an artificial grammar with materials generated from the Bohlen-Pierce scale, Loui and colleagues found that learning is impaired after small intervals are removed from the melodic structure [55]. Further Loui and colleagues argued that neural substrates of incidental grammar acquisition are independent of intelligence, pitch discrimination, pitch memory, musical training or working memory [56], [57]. Another study by Creel et al [58] showed that nonlocal interleaved tone triplets (of the form AxByCz) could only be learned when their pitches were separated. This suggests an interaction with streaming processes [59] that made both substructures be processed separately.

The lack of significant differences between musicians' and nonmusicians' performance in the present study suggests, similarly to the findings by Rohrmeier and colleagues and Loui and colleagues [15], [17], that the advantage of intensive training in and interaction with Western music has little impact once artificial stimuli which violate common melodic principles are employed, supporting a view that a core learning mechanism is involved. Further, the finding that participants knew to some extent when they were giving right responses suggests that they learned the structures well enough to possess explicit judgment knowledge and accords with the findings in the baseline study [15]. This does, however, not entail that the participants acquired explicit knowledge of the rules underlying the melodic system. It rather is analogous to the case in which a native English speaker may be entirely confident that a probe sentence is ungrammatical, yet may not be able to give an explicit account of the underlying grammatical rule that was violated. Hence the present results do not entail the participants formed explicit notions about the artificial grammar or other underlying rules.

In general, experimental findings of this study provide a case of how artificial grammar learning is affected by prior factors and reinforces the idea that prior entrenched structures are better learned incidentally than novel irregular structures. There have been discussions of the possibility that implicit learning could interact with prior knowledge (e.g. [60]–[63]). Several accounts of empirical evidence can be related this this: In their music experiment with serialist transformations, Dienes and Longuet-Higgins found that only a highly experienced expert participant could implicitly acquire serialist melodic transformations whereas inexperience participants performed at chance [21]. This implies an interaction between prior experience or prior-established processing pathways and the learning of novel complex structure. In an artificial grammar learning experiment, Perruchet and Peeremans found a marginally significant effect of the letter set (low vs. high frequencies in the participants' native language) [64]. Two studies showed effects on constraints of implicit learning with respect to form-meaning connections: participants could learn a linguistically meaningful variable (animacy), but not an arbitrary relation without linguistic relevance (relative size) [65], [66]. When participants had to learn sequences of cities (instead of letters) as potential travel routes, prior knowledge about the distances between cities facilitated or inhibited implicit grammar learning depending on plausible or implausible travel routes [67]. Similarly, using highly meaningful materials, unlike most other implicit learning studies, Ziori and Dienes found that prior knowledge facilitated implicit learning and resulted in a higher performance than for unrelated materials [60]. Prior knowledge gated learning performance in the context of category learning (cf. [68], [69]).

On the other hand, the impact of processing constraints on implicit or statistical learning still requires further research. Shukla and colleagues showed an interaction between statistical structures and prosodic features: strings of three syllables featuring high transition probabilities are not identified as words when they violate prosodic constraints [70]. In addition, Onnis and colleagues found that phonological features have an impact on the statistical learning of segmenting continuous speech into words [71]. However, many computational models of implicit learning do not incorporate effects of (pre-)processing or prior knowledge [62], [72]–[76], though there are exceptions such as [77] or [61]. Finally, Altmann showed that pre-training of a Simple Recurrent Network [78] with similar stimulus materials made it possible to model infant grammar learning [79] with a SRN although other modelling attempts had failed [80]. He explained the result in terms of pre-training as having avoided catastrophic interference of training items with items learned during the testing.

In the context of this general background, there are several potential explanations of the findings of the present study. One explanation is that the small interval fragments that constitute the building blocks for the stimuli are untypical and infrequent in common Western melodies. This may impede the ease of their recognition or priming, which in turn increases the cognitive processing load involved and consequently may affect their integration into higher-order chunks and larger sequences (cf. [76]). Such an explanation would be a counterpart to the finding by Scott and Dienes that prior familiarity with building blocks enhances implicit artificial grammar learning [81]. This explanation may be independent of whether such a difference in processing may be accounted for in terms of chunk probabilities or Gestalt principles.

Another potential explanation would be interference with streaming: Through the frequent violation of Narmour€s melodic principles the materials contain a large number of melodic leaps and large intervals (although the range is limited to 15 semitones). Accordingly the processing of these melodies may interfere with melodic streaming processes (cf. [59], [82]) so that the melodic sequences are not coherently (or not easily) processed as one single stream. In consequence, processing, recognition and learning of melodic chunks may be impaired.

Finally, one might explain the impaired performance in terms of mere statistical learning (e.g. [7], [82], [83]). First, since the underlying grammar in both experiments is the same and the surface sequences have matching n-gram structures, one might not expect a difference in performance merely in terms of statistical learning. However, this prediction changes when taking into account another assumption that the statistical learner comes endowed with a body of fragment knowledge from large exposure with common melodies. This knowledge adds a prior to the model that is likely to have a negative impact on the performance in the learning experiment since both, grammatical and ungrammatical stimuli are expected to be processed as unlikely (and less distinct) when dealing with Narmour-inconsistent materials. Hence mere statistical learning may provide another potential explanation of the current results.

Whether the difference in performance is due to the impedance of ease of processing, streaming or statistical learning biased by prior knowledge cannot be immediately answered from the present data. This therefore raises the question of whether or not these behavioural results can be accounted for through a simulation with computational models of statistical and implicit learning. One may hypothesise that the third explanation based on statistical learning with prior knowledge is the simplest since it provides the most simple cognitive assumptions (without additional assumptions about streaming, Gestalt principles or the like). If such computational models would fail to explain the challenge of the present reference results, a more complex underlying cognitive process need be assumed. This hypothesis remains to be addressed in future work.

The finding that uncommon melodic structures are less well learned may raise another point concerning melodic structure and Narmours principles: Do melodic structures following Narmours principles in general afford for better learnability or are they just learned better because they are more common? Melodic structures are largely found to follow Narmours principles across cultures and styles. Although the results by Pearce & Wiggins showed that a series of experimental studies on melodic perception [34], [37], [84] could be better explained by corpus-based statistical learning and processing rather than Narmours principles [35], [83], their finding does not entail why melodies accord with Narmours principles across cultures. In contrast, from an unbiased statistical learning perspective one might expect that melodic patterns (of the same complexity) are learned equally well independently of whether they accord with Narmours principles since a pure statistical processor would be indifferent to this distinction.

This cross-cultural convergence as well as the findings of impaired learning after violating Narmours principles in the present study as well as by [55] seems to suggest that ease of processing and learning constitutes a selective pressure for the (historical) change and emergence of melodic structures. Accordingly, factors of performance (such as preprocessing or streaming) may affect melodic learning, representation and reproduction and result in shaping the structures of melodies in larger timescales in a way analogous to the effect of performative constraints on grammars in language [85]–[87]. One may consequently understand implicit learning and its constraints as a *bottleneck* (grounding in communicative pressure, cf. [86], [88] ) for the learning, recognition, representation and reproduction of melodic structures which plays a significant role for the stabilisation and emergence of melodic structures [7], [89]–[93].
